# A Scoping Review on Biomarkers of Endothelial Dysfunction in Small Vessel Disease: Molecular Insights from Human Studies

**DOI:** 10.3390/ijms241713114

**Published:** 2023-08-23

**Authors:** Daniela Jaime Garcia, Audrey Chagnot, Joanna M. Wardlaw, Axel Montagne

**Affiliations:** 1 Centre for Clinical Brain Sciences, University of Edinburgh, Edinburgh EH16 4SB, UK; dany.jaime@ed.ac.uk (D.J.G.); joanna.wardlaw@ed.ac.uk (J.M.W.); 2 UK Dementia Research Institute, University of Edinburgh, Edinburgh EH16 4SB, UK; audrey.chagnot@ed.ac.uk

**Keywords:** endothelial dysfunction, endothelial cells, endothelial activation, small vessel disease, vascular dementia, blood–brain barrier, cerebrovascular dysfunction, white matter hyperintensities, lacunar stroke, lacune, microbleeds, perivascular spaces

## Abstract

Small vessel disease (SVD) is a highly prevalent disorder of the brain’s microvessels and a common cause of dementia as well as ischaemic and haemorrhagic strokes. Though much about the underlying pathophysiology of SVD remains poorly understood, a wealth of recently published evidence strongly suggests a key role of microvessel endothelial dysfunction and a compromised blood–brain barrier (BBB) in the development and progression of the disease. Understanding the causes and downstream consequences associated with endothelial dysfunction in this pathological context could aid in the development of effective diagnostic and prognostic tools and provide promising avenues for potential therapeutic interventions. In this scoping review, we aim to summarise the findings from clinical studies examining the role of the molecular mechanisms underlying endothelial dysfunction in SVD, focussing on biochemical markers of endothelial dysfunction detectable in biofluids, including cell adhesion molecules, BBB transporters, cytokines/chemokines, inflammatory markers, coagulation factors, growth factors, and markers involved in the nitric oxide cascade.

## 1. Introduction

Small vessel disease (SVD) is a highly prevalent disorder of the brain’s perforating microvessels. It is the underlying cause of a quarter of all ischaemic strokes and most haemorrhagic strokes and is the commonest cause of vascular dementia, contributing to approximately 45% of all dementias worldwide [1]. Radiological signs of SVD include lacunes, small subcortical infarcts, white matter hyperintensities (WMH), microbleeds, brain atrophy, and enlarged perivascular spaces (PVS), and are clinically associated with lacunar stroke, cognitive decline, neuropsychiatric symptoms and mobility problems [2,3,4,5]. Despite its increasing incidence and importance in the public health domain, the underlying pathophysiology of SVD remains poorly understood, and there is currently no effective therapeutic intervention beyond secondary stroke-prevention strategies.

SVD is associated with cerebral vascular dysfunction, characterised by structural and functional alterations in cerebral blood vessels, in blood flow responsiveness to changing metabolic demands, and a compromised and permeable blood–brain barrier (BBB), which precedes visible brain lesions [6]. The BBB is a highly specialised and dynamic structure referring to the set of characteristics in the vessels that vascularise the brain. It is formed by a tight monolayer of endothelial cells that line the blood vessel lumen, which uses selective permeability to closely regulate the exchange of substances between the circulating blood and the brain. Its function is also influenced by the constituents of the neurogliovascular unit, including pericytes, astrocyte endfeet, microglia, oligodendrocytes, the basal membrane, and neurons. Optimal BBB functioning is required to maintain healthy brain function by allowing for the adequate delivery of nutrients and oxygen to neurons, promoting drainage of metabolic waste, and protecting neurons from pathogens and neurotoxic compounds [7,8].

Brain endothelial cells are joined by tight junctions and serve to maintain the integrity of the BBB as well as play a role in modulating vascular tone, blood flow, inflammation, thrombosis, and immune cell adhesion and transmigration [7,8,9,10]. Endothelial cells are responsive to changes in their environment and can sense and adapt to fluctuations in blood flow or pressure through surface mechanoreceptors as well as communicate with their cellular environments through ion channels and signalling molecules and proteins that can alter their signature and thus influence brain vessel function [11,12]. Endothelial malfunction can be detected in vivo by quantifying soluble blood and cerebrospinal fluid (CSF) markers that are released into the circulation and offer a window into the diverse facets of endothelial function that contribute to homeostasis; these include cell adhesion molecules, BBB transporters, cytokines and chemokines, pro-inflammatory markers, coagulation factors, growth factors, and markers pertaining to the nitric oxide (NO) pathway.

In the pathological context of SVD, and indeed to an extent in normal ageing, there is a loss of some of the properties that maintain BBB integrity and a propensity towards endothelial dysfunction and activation, both of which carry downstream consequences influencing the function of surrounding cells and tissue and are associated with disease burden and progression [4,5,6,13,14,15,16,17,18,19,20,21,22,23]. Within this review, the term ‘endothelial dysfunction’ encompasses the established definitions of both endothelial dysfunction, marked by decreased endothelium-derived nitric oxide activity, and endothelial activation, which refers to the increased expression of endothelial cell-surface adhesion molecules and leukocyte adhesion molecules induced by pro-inflammatory cytokines [10,24]. Though there is mounting evidence advocating for the role of endothelial dysfunction in SVD, the underpinning molecular mechanisms and better methods to detect dysfunction in vivo remain unclear. In this scoping review, we aim to summarise the findings from clinical studies examining the role of the molecular mechanisms underlying endothelial dysfunction and activation in SVD, focussing on biochemical markers of endothelial dysfunction detectable in blood or CSF.

## 2. Methods

### Literature Review

We reviewed the literature examining biomarkers pertaining to endothelial dysfunction and radiological features of SVD in clinical studies. The literature search was conducted using MEDLINE and EMBASE from database inception to 1 June 2023 using key terms relating to small vessel disease (lacunes, small subcortical infarcts, white matter hyperintensities, microbleeds, and enlarged perivascular spaces) combined with terms relating to the endothelium (endotheli*). All articles were screened for eligibility criteria. We included publications relating to humans and examining biochemical markers of endothelial dysfunction detectable in blood or CSF and known radiological markers of SVD. We excluded review articles/meta-analyses, publications relating to study rationale and design, publications based exclusively on case studies or monogenic forms of SVD (including Cerebral Autosomal Dominant Arteriopathy with Sub-cortical Infarcts and Leukoencephalopathy (CADASIL)), as well as articles solely pertaining to mechanisms of cardiovascular or large-artery disease (e.g., coronary artery disease, atherosclerosis). Duplicate articles were removed. Additional studies examining the relationship between radiological signs of SVD and biomarkers of endothelial dysfunction from reference lists were also manually added to reinforce the search. We extracted summary data for each paper, including main aims, study population and sample size, methods employed, adjustment for covariates and primary findings.

## 3. Results

**Figure 1:**
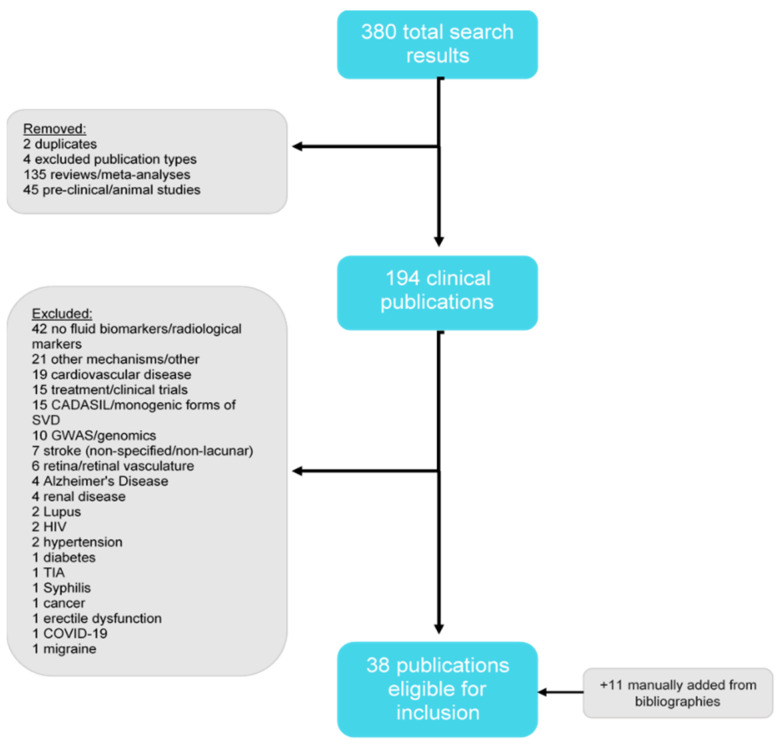
Study screening and selection process. A total of 49 publications were included in this review, including 11 manually selected from bibliographies.

The search yielded 380 results, 135 of which were classified as reviews and were thus removed, as were two duplicate publications and four papers pertaining to excluded publication types (Figure 1). Of the 239 remaining results, 194 publications described clinical studies, of which 38 were eligible for inclusion based on the criteria outlined above. In addition, 11 papers were manually added from reference lists.

## 4. Biomarkers of Endothelial Dysfunction and SVD: Molecular Insights from Human Studies

### 4.1. Immunoglobulin Superfamily—Cell Adhesion Molecules and Selectins 

In a homeostatic state, most cell adhesion molecules are constitutively expressed at low levels in the brain vasculature, and endothelial cells have a limited adhesiveness to leukocytes. However, a cytokine-activated endothelium can express cell-surface adhesion molecules that facilitate the recruitment, rolling, adhesion and transendothelial migration of leukocytes through the endothelium of vascular walls; the soluble forms of these molecules are released from the cell surface into the circulation and can serve as biomarkers of endothelial activation [25,26,27]. Endothelium-leukocyte interactions engender a cyclic activation of both components, leading to the production of neopterin and cytokines, which can also instigate hepatic production of C-reactive protein (CRP), thus exacerbating an inflammatory response [28,29]. There is also evidence suggesting the involvement of other constituents of the neurogliovascular unit, particularly pericytes, in dysfunctional crosstalk, which can further aggravate the endothelium [13].

Vascular cell adhesion molecule-1 (VCAM-1) and intracellular adhesion molecule-1 (ICAM-1) are transmembrane glycoproteins from the immunoglobulin gene superfamily which bind to integrins on the cell surface, whilst endothelial–leukocyte adhesion molecules, Endothelial-selectin (E-selectin) and Platelet-selectin (P-selectin), interact with leukocyte surfaces via carbohydrate structures [27]. Circulating cell adhesion molecules have been linked to ageing and a series of age-related neurodegenerative disorders, including Alzheimer’s Disease (AD) and SVD, and there is substantial evidence suggesting their expression can lead to downstream signalling events that are associated with increased BBB permeability [22,30,31,32,33,34,35,36]. For instance, cultured human brain microvascular endothelial cells exposed to soluble VCAM-1 (sVCAM-1) exhibit increased permeability of a soluble tracer accompanied by evidence of actin stress fibre formation and a decrease in tight junctions [31].

We found 19 published studies investigating the relationship between cell adhesion molecules and radiological features of SVD, featuring a total of 7364 study participants (average sample size: 368.2).

In a study with 110 lacunar stroke patients and 50 community controls with similar vascular risk-factor profiles, those with lacunar stroke had higher levels of circulating ICAM-1 [18]. These associations remained significant after controlling for conventional vascular risk factors and were not found to be associated with age, sex, blood pressure, diabetes, myocardial infarction, or time from vascular event to blood draw (median: 210 days). They also found comparable ICAM-1 concentrations amongst patients with isolated lacunar infarcts and those with WMH lesions.

Similarly, circulating levels of ICAM-1, VCAM-1 and P-selectin (though not E-selectin) were significantly higher in a group of 163 first-ever lacunar stroke patients compared to essential hypertensive patients and healthy controls [34]. Those with extensive WMH lesions had higher concentrations of sVCAM-1 than those with mild or absent WMH, and those with radiological evidence of both, a lacunar infarct and WMH lesions had the highest levels of sVCAM-1 and sICAM-1 on average. Though a marginal portion of the essential hypertensive group was found to have asymptomatic lacunes (n = 13/183) and an extensive WMH burden (n = 22/183) on magnetic resonance imaging (MRI), the distinct biomarker signatures between these groups provide further evidence in support of SVD-specific pathology beyond hypertension-related vascular injury. In the same study, multivariate analyses controlling for age, sex, pre-existing large-artery disease, and patient group revealed a positive association between higher levels of sE-selectin and the number of microbleeds, perhaps suggesting a haemorrhagic phenotype-specific role. This is further supported by another study with 126 first-ever ischaemic stroke patients, where higher levels of sE-selectin within 24 h of hospital admission were associated with the prevalence of microbleeds after adjusting for demographic and clinical variables [37]. No significant differences in P-selectin-positive platelets were found amongst 74 lacunar stroke patients and 74 controls matched for age, sex, diabetes and hypertension status with no history of stroke, though the possible presence of radiological signs of SVD in controls was not ruled out, nor was the extent of SVD radiological burden taken into account in these analyses [38].

We found few studies examining the relationship between fluid biomarkers and enlarged PVS, though, in 100 mild ischaemic stroke patients, PVS burden in the basal ganglia was not associated with ICAM-1 levels. While this might indicate different pathological processes involved in the appearance of PVS, further investigation is needed [39].

Including mild ischaemic stroke patients as controls can help parse out potential effects independent of those stemming from an acute ischaemic event or large-artery pathology. Regression analyses in a group of 263 thrombolysed acute ischaemic stroke patients showed that a combined score comprised of the number of lacunes and WMH lesion severity as detectable on computerised tomography (CT) was associated with increased levels of sICAM-1 and sVCAM-1 at 90 days post-stroke after adjusting for age, sex, and vascular risk factors [40]. Interestingly, levels of these biomarkers were comparable between patients with WMH, cerebral atrophy and/or lacunes compared to those without at the time of stroke-symptom onset, suggesting possible fluctuations in circulating cell adhesion markers during an acute stroke phase. Compared to a group of 67 age- and sex-matched controls, 64 SVD patients and 109 patients with large-artery atherosclerosis had higher concentrations of sE-selectin and sICAM-1 (though not sVCAM-1), with the highest concentrations found in patients with intracranial macroangiopathy [41]. However, these analyses did not account for clinical covariates despite substantial group differences- for instance, nearly half of the patients with large-artery atherosclerosis were smokers, compared to 13% of SVD patients. In addition, some participants in every group had a history of stroke, including 21% of controls, which may have affected the results.

In a small study of 29 patients with SVD and 18 patients with recent ischaemic stroke on the basis of internal carotid artery stenosis, levels of sVCAM-1 and sP-selectin were significantly higher in patients in the highest quartile of periventricular WMH lesion burden compared to those in the lower quartiles [22]. This relationship did not extend to subcortical white matter lesions. To account for potential differences relating to the stroke itself, the authors performed a sub-analysis comparing cell adhesion molecule concentrations with degree of white matter lesions separately in patients with acute stroke and those with carotid atherosclerosis. Their results indicated that the increased levels of sVCAM-1 and sP-selectin in those with severe periventricular WMH lesions in the group were driven by the subgroup of patients with carotid atherosclerosis. It is plausible that this is a result of compounded chronic vascular damage, which might attenuate the relationship between WMH and cell adhesion molecules. It is important to note that cell adhesion molecule plasma concentrations in this small study sample were highly variable, and the dichotomisation of white matter lesions to ‘mild’ (including the lowest three quartiles of WMH lesion burden) and ‘severe’ (including the highest quartile), could potentially overlook a nuanced relationship between WMH volume and endothelial activation. In addition, though the study was comparing symptomatic SVD with large-artery atherosclerosis, both groups had comparable levels of WMH lesion burden, and the analyses did not account for potential confounding variables, including vascular risk factors.

Similar results are mirrored in studies with healthy ageing cohorts. In 175 healthy adults ≥60 years old with no history of stroke or cognitive impairment, those in the highest quartile of sICAM-1 levels had a four-fold increased risk of having WMH lesions on MRI after adjusting for age, sex and traditional vascular risk factors [42]. In a large Chinese community-based cohort study (n = 960; average age: 56.02), grouped endothelial dysfunction biomarkers, including sE-selectin, sP-selectin, sICAM-1 and sVCAM-1, were associated with WMH volume and the presence of lacunes, though not microbleeds or PVS, independent of age, sex and vascular risk factors; backward elimination regression retained sVCAM-1 as an independent predictor of WMH volume [43]. In the Framingham Offspring cohort, analyses with 1763 stroke-free individuals (average age: 60.2) showed higher levels of sICAM-1 (though not sP-selectin) in those with radiological signs of SVD, defined as the presence of lacunes and/or extensive WMH lesions on MRI. Though these results survived additional adjustment for clinical covariates, they did not withstand Bonferroni correction for multiple comparisons. It is worth noting that the mean time from blood draw to MRI was substantial, averaging 5.4 years, and thus it is possible that biomarker profiles were not representative of temporal disease progression. In addition, the study excluded those with a history of stroke, limiting the participation of individuals with symptomatic or more severe SVD; indeed, only 12–21% of participants had lacunes or extensive WMH lesions [44].

Longitudinal studies also support the involvement of cell adhesion molecules in SVD progression and adverse clinical outcomes. In a 10-month follow-up study, 35 SVD patients receiving a monthly MRI were stratified into those with SVD progression and those without, based on the appearance of incident lacunes, microbleeds or an increase in WMH volume. After adjusting for age, sex, and hypertension, circulating levels of sVCAM-1 and sE-selectin were higher in those with SVD progression compared to those without, though only the latter was statistically significant [45]. Changes in sICAM-1 and sVCAM-1 levels were positively associated with an increased risk of SVD progression in a 69-month longitudinal study with 1396 participants and were independently associated with WMH grade and the number of new appearing lacunes and microbleeds [46]. Likewise, amongst a cohort of 123 SVD patients followed-up at 2 years, those with radiological disease progression had a higher compound z-score comprised of markers of vascular inflammation, including sICAM-1 and sP-selectin, after adjusting for age, sex, blood pressure and total SVD radiological load [47]. Circulating levels of sICAM-1 were significantly higher in 113 acute lacunar stroke patients compared to 43 healthy controls [48]. Although potential effects stemming from an acute ischaemic event cannot be discounted, they also found that within those with a lacunar stroke, sICAM-1 levels were higher in those who had neurological deterioration within 48–72 h post-stroke (defined as a ≥ 1 point fall in the motor items of the Canadian Stroke Scale) than those with non-progressive strokes. After adjusting for potential confounding variables, sICAM-1 levels were also independently associated with poor outcomes 3 months post-stroke, including lower functional independence and death, though infarct volume and radiological disease burden could have mediated this association. Intriguingly, in an 8-year longitudinal cohort study of 495 cognitively unimpaired elderly individuals (average age: 70.02) and 247 participants (average age: 70.89) with mild cognitive impairment (MCI), CSF levels of ICAM-1 and VCAM-1 had independent effects on declining cognition, even after adjusting for WMH volume, potentially suggesting a distinct pathway involved in cognitive decline [49].

In the Austrian Stroke Prevention Study cohort, 296 community-dwelling participants received an MRI at baseline and again at 3 and 6 years [50]. After adjusting for age, sex, CRP levels, baseline WMH volume and traditional vascular risk factors, WMH lesion progression at both follow-up time points was associated with higher levels of circulating sICAM-1. In a longitudinal study of 190 type-2 diabetes patients, baseline levels of sICAM-1 were higher in patients with radiological signs of SVD than in those without and were also associated with the increased presence of incident lacunes and WMH lesion progression at 3 and 6 years after adjusting for age, sex, hypertension, duration of diabetes, baseline MRI findings and medication use [51]. They also demonstrated a significant trend association between higher levels of sICAM-1 and lower scores in a letter-digit psychomotor task, though this could be mediated by radiological disease burden.

Examining brain-endothelial specific molecular mechanisms of SVD in patients represents a significant challenge, and while quantifying biomarkers from circulating blood can reflect possible dysregulated pathways, it provides an indirect path of examination. Studies zoning in on brain endothelial cell signatures from SVD patients provide some conflicting evidence. A post-mortem study examining vessels from 17 SVD cases and 12 aged controls found little evidence of positive endothelial ICAM-1 vessels in either group [52]. The authors hypothesise that elevated circulating levels of sICAM-1 in SVD patients are, therefore, unlikely brain-derived and may indicate inflammatory activity in distal peripheral vascular territories. However, this study limited analyses to one cell adhesion molecule and small arteries within the caudate putamen grey matter areas, excluding other vascular territories directly impacted by SVD, namely the capillaries feeding into the white matter. While it remains feasible that high sICAM-1 levels in SVD patients could stem from beyond the brain endothelium, ICAM-1 is a heterogeneous molecule and can influence endothelial function through dysfunctional crosstalk involving other cells in the brain that can also derive it, including monocytes, macrophages, smooth muscle cells and pericytes [53]. Elahi et al. [54] used plasma membrane-derived vesicles released by cells (plasma endothelial-derived exosomes (EDE)) in 26 functionally intact older adults (average age: 75.4) to compare protein cargo between those with and without WMH lesions on MRI. Though SVD cases showed higher levels of brain-expressed EDE cargo proteins, and these were associated with lower cognitive function, there were no significant group differences detected in VCAM-1 levels specifically after adjusting for age.

Although there is evidence in support of the involvement of cell adhesion molecules in diverse manifestations of SVD, the lack of clarity on the underlying mechanisms through which they might influence disease and where the molecules derive from and how they engender and interact with pathological mechanisms warrants further examination.

### 4.2. Blood–Brain Barrier Transporters

Membrane transport proteins at the BBB mediate the influx and efflux of solutes from the circulation through endothelial cells and into the brain and vice-versa. Glucose transporter-1 (GLUT-1) is expressed in brain vascular endothelial cells and transports glucose to the brain via facilitative diffusion. GLUT-1 levels are implicated in metabolic dysregulations and are known to decrease with ageing, possibly disturbing neurovascular coupling processes based on glucose demand [55]. Permeability-glycoprotein (P-GP) is an adenosine triphosphate binding cassette transporter expressed by the luminal surface of endothelial cells. It is known for its role in pharmacokinetics and drug-delivery to the BBB by strictly limiting the exposure of pharmaceuticals to the brain by actively transporting undesired substrates from the BBB back to the bloodstream against the concentration gradient; it has also been implicated in the accumulation of amyloid-β (Aβ) in AD pathology [39,56]. Large neutral amino acid transporter-1 (LAT-1) is expressed in both the luminal and abluminal membranes of endothelial cells and carries large essential amino acids across the BBB [57]. Caveolin-1 (Cav-1) is the principal scaffold protein comprising caveolae, which are invaginations abundant on the cell membrane of endothelial cells, facilitating vesicle trafficking and signal transduction. Cav-1 activity is related to oxidative stress, the attenuation of inflammatory pathways, and BBB permeability, perhaps through the modulation of tight junction-associated proteins [58,59]. Pro-inflammatory cytokines can also upregulate BBB transporter protein expression in endothelial cells, affecting vascular function. For instance, increases in GLUT-1 in murine smooth muscle cells can impair vascular contractility and instigate an inflammatory response to vascular injury [60]. GLUT-1 deficiency in mice overexpressing Aβ-peptide precursor protein leads to significant reductions in blood flow, BBB and microvascular breakdown, accelerated Aβ accumulation, cognitive deficits and progressive neurodegeneration [61].

In an attempt to circumvent the low specificity of fluid biomarkers for the assessment of endothelial dysfunction, a case-control study with 26 functionally intact older adults (average age: 75.4) used endothelial-derived biomarkers to examine the relationship between WMH lesions and endothelial-expressed BBB transporter proteins GLUT-1, P-GP and LAT-1 [54]. EDE cargo protein quantification offers a non-invasive way of observing cellular and molecular changes in vivo using exosomes, which are cell-released vesicles containing cargo molecules such as proteins, mRNAs and lipids from their cell of origin. Levels of GLUT-1, LAT-1 and P-GP were significantly higher in participants with WMH than in those without. Age-adjusted logistic regression analyses demonstrated a robust predictive power of GLUT-1, LAT-1 and P-GP to differentiate between participants with and without WMH, with the area under the curve for the strongest predictor, GLUT-1, being 0.89. LAT-1 levels were significantly inversely associated with total grey matter volumes after controlling for intracranial volume (ICV) and age and correcting for multiple comparisons. In addition, all brain-expressed EDE cargo proteins were associated with lower cognitive performance in a set-shifting speed task after adjusting for age. Cytokines and chronic states of inflammation could up-regulate some BBB transporter proteins in endothelial cells, and interestingly, levels of GLUT-1, P-GP and LAT-1 were associated with levels of systemic inflammatory cytokine Interleukin-6 (IL-6) in this cohort [54].

In 156 first-ever ischaemic stroke patients, circulating Cav-1 levels were lower in patients with microbleeds compared to those without and lowest in patients with multiple microbleeds or microbleeds in deep or infratentorial areas compared to those with a single microbleed or strictly lobar microbleeds [62]. Patients below their established Cav-1 concentration cut-off (≤5.25 ng/mL) had a nearly four-fold increased risk of having microbleeds after adjusting for age, sex, vascular risk factors, anti-thrombotic use, glucose levels, lipids and fibrinogen. There was no relationship detected between Cav-1 levels and WMH lesions or lacunes.

Clinical research studies investigating the role of transport proteins in SVD pathogenesis are scarce. However, transporter proteins play a crucial role in BBB homeostasis and can potentially serve as promising targets to ameliorate the BBB dysfunction observed in the early stages of the disease. The studies mentioned here suggest a relationship between transporter protein dysregulation and some radiological manifestations of SVD, though more studies are required to validate and further uncover the nature of this relationship.

### 4.3. Cytokines and Chemokines

#### 4.3.1. Interleukin-6

IL-6 is a pro-inflammatory cytokine with pleiotropic effects produced by monocytes and endothelial cells. Once synthesised in a lesion site, it migrates to the liver, where it incites the release of several acute-phase proteins, including CRP and fibrinogen [63]. It also instigates platelet production and is thus involved in the development of atherosclerosis. Pre-clinical studies suggest IL-6 can engender endothelial dysfunction through an increase in Angiotensin II type 1 (AT1) receptor activity, an interaction with tumour necrosis factor alpha (TNF-α), and the induced production of reactive oxygen species (ROS), which can cause enhanced vascular superoxide production, exacerbate oxidative stress, and impair endothelium-dependent vasodilation [64,65]. Numerous clinical studies have examined the association between IL-6 and cardiovascular health, particularly in stroke, with many lines of evidence suggesting it serves as a strong predictor for stroke, atherosclerosis and adverse cardiovascular events [66,67].

In a Japanese study with 194 neurologically asymptomatic participants at a high risk of stroke, mean serum levels of IL-6 were higher in patients with lacunes visible on MRI than in those without, with one standard deviation increase in IL-6 leading to a nearly two-fold increased risk of having at least one visible lacune after adjusting for age, sex, vascular risk factors, medication use and carotid atherosclerosis [68]. In the Cardiovascular Health Study, both white (n = 3073) and black (n = 571) participants with higher IL-6 levels had a mildly increased risk of having WMH lesions and brain infarcts independent of age, sex and cardiovascular risk factors [69]. Likewise, in 247 elderly participants with MCI, though not in 495 cognitively unimpaired participants, baseline CSF IL-6 levels were associated with greater WMH volume after adjusting for age, sex, ICV and vascular risk factors [49]. Adjusting for CSF Aβ-40 attenuated the relationship. Intriguingly, lower IL-6 levels were associated with increasing WMH burden over 7 years in the same study. It is possible that the greater incidence and severity of WMH lesions in the MCI group increased the likelihood of finding significant relationships within this group due to enhanced statistical power.

In univariate analyses, plasma IL-6 concentrations were significantly higher in 113 lacunar stroke patients within 24 h of stroke onset compared to 43 healthy controls, though the possible confounding effects of an acute ischaemic event, demographic characteristics or comorbidities were not taken into account in these analyses [48]. In this same study, after adjusting for potential confounding variables, there was no association found between circulating levels of IL-6 and worsening clinical progression or poor outcome 3 months post-stroke. A study comparing 65 lacunar stroke patients with 60 cortical stroke patients reported no significant differences in circulating IL-6 levels between the groups, possibly suggesting the association between IL-6 and recent stroke might not be specific to SVD pathology [70]. It is important to consider that medication use (including aspirin treatment), glutamate and GABA levels, the presence or timing of stroke, and the degree of large-artery atherosclerosis are all elements that could influence circulating pro-inflammatory biomarker levels.

In line with previous research examining the relationship between IL-6 levels and cardiovascular event risk, IL-6 concentrations were associated with an increased risk (OR: 1.4, 95% CI: 1.1–2.2) of adverse vascular events, including stroke, cardiovascular events and/or vascular death in a 24-month longitudinal study with 130 functionally independent SVD patients [71]. This association was further confirmed by findings from the LIMITS study, which observed an association between elevated levels of circulating IL-6 and the risk of recurrent ischaemic stroke, myocardial infarction or vascular death in 1244 radiologically confirmed lacunar stroke patients [72]. However, a small 10-month longitudinal study with 35 SVD patients reported no significant association between baseline IL-6 levels and the appearance of new lesions on diffusion-weighted MR imaging, incident lacunes and/or microbleeds or WMH progression after adjusting for age, sex and hypertension [45].

Although studies examining the relationship between IL-6 and other radiological markers of SVD were lacking, one study reported finding no association between IL-6 and the presence of basal ganglia PVS on MRI after adjusting for potential confounding variables in 100 mild ischaemic stroke patients, both lacunar and cortical [73].

#### 4.3.2. Tumour Necrosis Factor Alpha and Its Receptors

TNF-α is a pro-inflammatory cytokine primarily secreted by macrophages and microglia that functions via cell membrane receptors, including tumour necrosis factor receptors 1 and 2 (TNFR1/TNFR2). TNF-α promotes the secretion of endothelial cell adhesion molecules, facilitating leukocyte adhesion and reduced blood flow [74]. High levels of TNF-α have been observed in patients with acute ischaemic stroke [75]. In both clinical and pre-clinical investigations, TNF-α has been associated with increased BBB permeability and dysfunction, inflammation, oligodendrocyte death and myelin damage [76,77,78]. In addition, increased levels of TNF-α can inhibit NO production in the endothelium, induce ROS production, and impair vasodilation [79,80].

Results from the LIMITS study provide some evidence of the relationship between TNFR1 and worse clinical outcomes in SVD patients. The risk of major vascular events, though not recurrent stroke, increased with elevated concentrations of TNFR1 (HR: 1.68, 95% CI: 0.93–3.04) after adjusting for demographics, risk factors and statin use in 1244 radiologically-confirmed lacunar stroke patients (average age: 63.3) [72]. There was no evidence that TNFR1 was associated with the risk of having a recurrent lacunar stroke.

Average plasma concentrations of TNF-α were significantly higher in 113 acute lacunar stroke patients compared to 43 healthy controls in univariate analyses [48]. In addition, within lacunar stroke patients, levels of TNF-α were higher in those who had neurological deterioration (defined as a fall of ≥1 point in the motor items of the Canadian Stroke Scale within inclusion and 48 h) and served as an independent predictor for worse clinical outcome (Barthel index < 85 or death). However, it is worth noting that the control group was considerably younger (average age: 55) compared to the lacunar stroke group (average age: 69.7). Moreover, comparing an acute-stroke group to a non-stroke group provides limitations in the interpretation of results, particularly as it relates to the variation of pro-inflammatory molecules that may fluctuate as a direct consequence to an acute ischaemic event. Indeed, results from another study reported no significant differences in circulating TNF-α levels between 60 cortical stroke patients and 65 lacunar stroke patients 1–3 months post-stroke [70].

Studies examining the relationship between TNF- α and radiological markers of SVD beyond WMH lesions are sparse. In a prospective study with 100 mild ischaemic stroke patients, there were no significant associations between TNF-α and PVS after adjusting for potential covariates [73]. In the Framingham Heart Study cohort (sample size: 1763, average age: 60.2), higher levels of circulating TNFR2 were associated with the presence of deep cerebral microbleeds after adjusting for age, sex, time from blood draw to MRI (average: 5 years), hypertension, WMH volume, number of lacunes, and medication use [44]. In this study, increases in TNFR2 related to a 3.3-fold increase in the odds of having more than one microbleed and a 5.7-fold increase in the odds of having more than two microbleeds, possibly suggesting distinct mechanistic pathways involved in ischaemic and haemorrhagic manifestations of SVD.

Dobrynina and colleagues [81] performed a cluster comparison in 40 SVD patients with a Fazekas score of three to categorise patients into two radiological subtypes of SVD. Patients in the MRI subgroup characterised by more severe deep WMH, lacunes in the white matter as opposed to dispersed, a lack of microbleeds or atrophy and less severe clinical manifestations had significantly higher levels of TNF-α compared to those in the other MRI group and healthy controls.

#### 4.3.3. Osteoprotegerin

Osteoprotegerin is a cytokine expressed by endothelial cells and smooth muscle cells involved in inflammation and vascular injury. High levels have been associated with large-artery atherosclerosis and stroke, though there is limited research regarding its relation to SVD [82]. Results from the Framingham Offspring cohort study (sample size: 1763; average age: 60.2) showed that participants with lacunes and/or extensive WMH lesions had higher levels of osteoprotegerin than those without after adjusting for age, sex, vascular risk factors and the presence of microbleeds; the relationship also withstood Bonferroni correction for multiple comparisons [44].

#### 4.3.4. Platelet Factor-4

Platelet factor-4 (PF-4) is a chemokine stored in the alpha granules of platelets. It is released on activation, whereupon is exerts angiostatic effects by inhibiting the binding of specific growth factors, such as vascular endothelial growth factor (VEGF), to cells [83]. PF-4 has previously been associated with stroke risk, infarct size and worse clinical outcomes post-stroke [84]. A prospective study featuring 123 SVD patients aged over 60 years demonstrated that patients with radiological disease progression, as measured by an increase in WMH severity, had higher levels of PF-4 after adjusting for age, sex, blood pressure and total SVD radiological load [47]. The precise mechanisms underpinning the interaction between SVD and PF-4 remain unclear, and larger studies are required to reach more robust and definitive conclusions.

### 4.4. Other Pro-Inflammatory Markers

#### 4.4.1. C-Reactive Protein

CRP is a hepatic acute-phase reactant protein known to increase in concentration as a response to IL-6 secretion and systemic inflammation. Though little is known about its direct role in SVD, there is an established connection between CRP and cardiovascular-event risk, including stroke [85,86]. CRP can influence endothelial dysfunction in conjunction with its involvement in systemic inflammation through a variety of diverse mechanisms. For instance, CRP activity can inhibit angiogenesis, promote atherosclerosis, mediate circulating cytokine levels, inhibit NO, decrease endothelial nitric oxide synthase (eNOS) activity in endothelial cells, increase Endothelin-1 production, attenuate endothelial-dependent vasodilation and increase endothelial cell adhesion molecule expression, including VCAM-1 and E-selectin [87,88,89].

We found 12 studies investigating the relationship between CRP levels and radiological signs of SVD, featuring a total of 9794 study participants (average sample size: 816.16).

Most studies featuring acute and sub-acute stroke patients do not report strong associations between CRP levels and SVD disease burden, though it is important to note that CRP levels are influenced by ischaemic events. For instance, baseline CRP levels were higher in 74 lacunar stroke patients within a month of symptom onset compared to 74 controls matched for age, sex, hypertension and diabetes; however, this difference was attenuated by 3 months post-stroke, possibly suggesting that the observed elevated CRP levels in lacunar stroke patients were attributable to an acute ischaemic event rather than chronic SVD disease mechanisms [38]. In a study with 65 lacunar stroke patients and 60 cortical stroke patients serving as controls, CRP levels did not vary significantly between the groups after adjusting for age, sex and the presence of traditional vascular risk factors. However, incorporating a cluster of inflammatory markers, including CRP, TNF-α and IL-6, into their base model for predicting WMH volume mildly improved model fit (from R2 = 0.289 to R2 = 0.291) [70].

Though WMH lesion burden and the presence of lacunes constituted the main outcomes in most of the publications we found, a study examining the association between PVS in the basal ganglia and circulating CRP levels at 1–3 months post mild cortical or lacunar stroke in 100 patients reported no significant associations after controlling for age, sex and vascular risk factors [73]. In addition, a recent study reported significantly higher levels of CRP in SVD patients with cognitive impairment (n = 40) compared to SVD patients without cognitive impairment (n = 38) and healthy controls (n = 35), respectively [90]. However, these analyses did not account for potential covariates and included a relatively small sample size.

In line with existing evidence, longitudinal studies with SVD patients support the relationship between baseline CRP levels and recurrent stroke. In 1422 lacunar stroke patients with no prior history of cortical stroke or carotid stenosis, patients in the highest quartile of circulating CRP levels measured in the subacute post-stroke phase (median: 60 days) had more than twice the risk of recurrent ischaemic stroke at 3-year follow-up (OR: 2.32, 95% CI: 1.15–4.68) after adjusting for age, sex, race-ethnicity, history of cardiac disease, vascular risk factors and statin use [91]. Although approximately 70% of recurrent ischaemic strokes were of lacunar aetiology, there was no evidence of a statistically significant predictive effect of CRP on recurrent lacunar stroke specifically. In addition, a small study with 130 SVD patients (including 28 vascular Parkinsonism patients) reported a marginal association between baseline CRP levels and recurrent stroke, death or cardiovascular events at 24-month follow-up after adjusting for age, sex, vascular risk factors, statin or antiplatelet use, and radiological SVD burden (HR 1.02, 95% CI: 0.98–1.7) [71].

Larger population-based studies with stroke-free participants have yielded conflicting results. Findings from the Rotterdam cohort (n = 1033, average age: 72) showed that those in the highest quartile of CRP levels at baseline had more extensive WMH lesions compared to those in the lowest quartile after adjusting for age, sex, cardiovascular risk factors and carotid atherosclerosis [92]. In the 636 participants who had a repeat MRI at 3 years, CRP levels at baseline were also associated with WMH lesion progression and number of new lacunes, though the latter association was not statistically significant. A small study with 194 neurologically asymptomatic patients with vascular risk factors showed similar results, with circulating levels of CRP being higher in those with lacunar infarcts compared to those without and CRP levels functioning as an independent predictor for lacunar infarcts after adjusting for risk factors and carotid atherosclerotic vascular disease (OR 1.85 per SD increase in CRP) [68]. Similarly, in 3644 participants (average age: 74.65) in the Cardiovascular Health Study, both white (n = 3073) and black (n = 571) participants with higher CRP levels had a mildly increased risk of having WMH lesions (OR: 1.13, 95% CI: 1.06–1.22) independent of age, sex and cardiovascular risk factors- though statistical significance was reached only in white participants. Plasma CRP levels were only marginally associated with the presence of brain infarcts in the two groups, though the infarct subtype was not specified. Moreover, there was no association found between CRP haplotypes or genotypes and the appearance of brain infarcts or WMH lesions [69].

Contrastingly, in 1763 stroke-free individuals in the Framingham Offspring cohort (average age: 60.2), there were no significant associations found between circulating CRP levels and the presence of lacunes, extensive WMH lesions or microbleeds [44]. In a longitudinal study of 190 type-2 diabetic patients, baseline levels of CRP were comparable between patients with radiological signs of SVD and those without [51]. In addition, there was no relationship between CRP levels at baseline and SVD progression at 3 or 6 years. However, previous research has shown an association between glycaemic control and systemic inflammation in people with diabetes, with increasing levels of haemoglobin A1c (HbA1c) being significantly associated with elevated CRP levels after adjusting for covariates [93]. Therefore, it is possible that CRP levels were elevated within the control group comprised of diabetic patients, and HbA1c levels were not incorporated into these analyses.

#### 4.4.2. Neopterin

Neopterin is a pteridine immune modulator produced by activated monocytes and macrophages and can serve as a marker for inflammation. Higher neopterin levels have been detected in patients with cardiovascular disease and are associated with an increased risk of stroke [94]. Due to endothelium-leukocyte interactions, neopterin may play a direct role in inciting endothelial activation, possibly by promoting cell adhesion molecule expression mediated by oxygen free radicals [29,95]. Research relating neopterin to SVD is lacking, though one study with 163 lacunar stroke patients and 183 essential hypertensive patients reported higher levels of circulating neopterin in patients with lacunar infarct and/or WMH lesions compared to those without [34]. In addition, neopterin levels were independently associated with a higher number of PVS in the basal ganglia after controlling for age, sex, pre-existing large-artery disease, and patient group.

#### 4.4.3. CD40 Ligand

CD40 ligand (CD40) is a transmembrane glycoprotein expressed by endothelial cells and activated T-cells in an inflammatory state. It has been implicated in the activation of endothelial cells and leukocyte adhesion; its anchoring to the endothelium not only encourages the production of pro-inflammatory cytokines but also enhances the expression of VCAM-1, ICAM-1 and E-selectin [96]. CD40 has previously been associated with stroke risk, infarct size and worse clinical outcomes post-stroke [84].

Findings from the Framingham Heart Study suggested no association between plasma CD40 levels and WMH, lacunes or microbleeds in 1763 stroke-free participants [44]. In a prospective cohort study with 123 SVD patients aged over 60, those with radiological disease progression at 2-year follow-up had a higher z-score comprised of markers of vascular inflammation, which included CD40 ligand, after adjusting for age, sex, blood pressure, and total SVD radiological load score [47]. However, there were no reported independent associations between CD40 and radiological features of SVD or disease progression. Moreover, analyses with 1244 lacunar stroke patients in the LIMITS study demonstrated no association between circulating levels of CD40 and recurrent ischaemic stroke (including lacunar stroke), myocardial infarction, or vascular death, though analyses exploring CD40 and radiological SVD burden were not performed as part of this study [72].

Though a wealth of evidence suggests the involvement of systemic inflammation in stroke and cerebrovascular dysfunction, it is important to consider certain elements when dissecting the discrepant results from these studies. Firstly, a variety of factors can influence circulatory inflammatory markers, including comorbidities, concurrent infection, malignancy, medication use, and rheumatologic disease. Though some of these can be plausibly controlled for in certain study conditions, it remains unclear whether circulating biomarkers of systemic inflammation can serve as an adequate proxy for brain endothelial dysfunction. MRI markers of SVD reflect evidence of an accumulation of chronic disease, whereas blood biomarkers can fluctuate rapidly in response to acute events or homeostatic changes, something that bears particular relevance in the acute and subacute stages of stroke. For this reason, it is important to note the variability in the time window between stroke onset, serology and MRI acquisition in stroke studies and whether the control group was comprised of non-lacunar stroke or non-stroke participants. In addition, CRP levels have a known association with atherosclerosis and other large-artery-related pathology, which not only shares a vascular risk factor profile overlap with SVD, but might also chronically exert effects on the microvasculature. Whether SVD could be partly caused by the release of pro-inflammatory molecules into the brain microvessels and cellular milieu or whether it induces the synthesis and release of these molecules also remains unclear.

### 4.5. Coagulation Factors

Physiological haemostasis is preserved through blood constituents, blood flow regulation and the integrity of the vessel wall. Endothelial cells play a crucial role in the maintenance of physiological haemostasis and the modulation of thrombosis through the tight control of both pro-coagulant and anti-coagulant mechanisms. Not only can the endothelium provide a surface for thrombosis formation, but it also regulates blood flow and mediates the expression of vasoactive factors relating to platelet reactivity, coagulation and fibrinolysis. At sites of vascular injury, platelets migrate to the endothelium and adhere to the injury site, where they form clots to effectively seal the barrier and prevent excessive blood loss. Several studies have investigated the relationship between circulating coagulation factors and radiological markers of SVD.

#### 4.5.1. Von Willebrand Factor

Von Willebrand Factor (vWF) is a large polymeric blood glycoprotein synthesised by endothelial cells. Although it plays a role in haemostasis and thrombus formation by sensing and responding to shear blood flow changes, one of its main functions is to facilitate platelet adhesion to the endothelium in sites of vascular injury. States of chronic inflammation or endothelial activation can encourage excessive secretion of vWF from secretory granules within dysfunctional endothelial cells and increase platelet binding to vWF. Furthermore, high levels of circulating vWF are associated with prothrombotic complications, diabetes, stroke, and inflammatory cardiovascular disease [97]. Though some evidence suggests plasma vWF can function as a biomarker of endothelial dysfunction, considerable inconsistencies remain among clinical studies [98].

Average vWF levels were higher in a group of 74 recent lacunar stroke patients compared to 74 controls matched for age, sex, hypertension and diabetes; however, vWF levels had significantly decreased by 3 months post-stroke and were at comparable levels between groups, perhaps suggesting that chronic vWF elevation was not playing a central role in SVD pathophysiology [38]. A study of 263 thrombolysed acute ischaemic stroke patients further supports these findings, with vWF levels found comparable between patients with WMH lesions, cerebral atrophy or lacunes visible on CT compared to those without at the time of stroke onset [40]. Adjusted analyses at 90 days post-stroke showed similar results. Likewise, a previous study reported that vWF levels were increased to a similar extent in patients of acute-phase lacunar stroke as in patients of large-artery atherosclerotic and cardioembolic strokes [99]. There were also no significant differences in vWF levels between 65 lacunar stroke patients and 60 cortical stroke patients 1–3 months post-stroke after adjusting for age, sex and vascular risk factor profiles [70]. Collectively, these studies provide evidence for the hypothesis that although vWF might serve as a marker of endothelial dysfunction consequent to an acute endothelial response to an ischaemic insult, it might not reflect any SVD-specific pathogenic effect.

Notably, some studies suggest that an active form of vWF can be distinguished from a latent form, and though the use of total circulating vWF has yielded conflicting results, it is possible that differentiating between these subtypes could prove insightful in the context of SVD [100]. The ability to control vWF secretion from endothelial cells might serve as a future therapeutic target, though more research, particularly in the form of large longitudinal studies, is required to adequately differentiate the role of vWF from an acute response to an ischaemic insult versus a marker of chronic endothelial dysfunction pertinent to SVD disease mechanisms.

#### 4.5.2. Tissue Factor and Tissue Factor Pathway Inhibitor

Tissue factor (TF) binds to coagulation factors and thrombin to form fibrin. TF is normally concealed within the intact vessel wall until it becomes exposed after vascular injury, wherein it initiates the clotting response with the recruitment of platelets and coagulation factors. Though it is most commonly expressed by perivascular cells and platelets, it can be secreted by the endothelium, particularly under pathophysiological conditions in which endothelial cell-secreted miR-126 inhibits thrombosis by attenuating TF expression [101]. Decreased levels of TF have been found in patients with ischaemic stroke, including lacunar stroke [102]. Tissue factor pathway inhibitor (TFPI) is an anticoagulation protein primarily secreted by endothelial cells, which can inhibit TF activity and thereby modulate thrombosis [103].

In the 6-year longitudinal neuroimaging Austrian Stroke Prevention Study, no significant associations were found between SVD features or disease progression and TFPI in 296 community-dwelling participants after adjusting for age, sex, CRP levels, baseline WMH volume and traditional cardiovascular risk factors [50]. Importantly, as this cohort was not an SVD cohort, WMH severity grading was ‘none’ or ‘punctate’ in the vast majority (86.1%) of the sample, with only 24 participants out of 259 being classified as having SVD progression by 3 years. Another study found that TFPI levels were higher on average after adjusting for traditional vascular risk factors in a group of 110 symptomatic SVD patients compared to 50 healthy controls [18]. Patients with widespread WMH lesions had lower TFPI levels and a higher TF/TFPI ratio compared to those with isolated lacunar infarction. These results suggest that endothelial prothrombotic changes may be important in mediating an ischaemic SVD phenotype.

#### 4.5.3. Tissue-Type Plasminogen Activator and Plasminogen Activator Inhibitor-1

Tissue-type plasminogen activator (t-PA) is a serine protease synthesised by endothelial cells that plays a critical role in fibrinolysis. It is associated with endothelial activation and increased BBB permeability [104]. Overexpression of t-PA is a known risk factor for future ischaemic stroke [105]. Plasminogen activator inhibitor-1 (PAI-1) is a serine protease inhibitor primarily produced by endothelial cells involved in the suppression of fibrinolysis through the inhibition of t-PA. Physiological levels of PAI-1 are required for haemostasis, and increased concentrations of PAI-1 are associated with atherosclerosis, thrombosis, cellular senescence and endothelial dysfunction [106,107]. Studies using in vitro BBB models provide further support regarding the involvement of t-PA in endothelial dysfunction, showing a dose-dependent effect on BBB permeability and endothelial cell morphological changes through the activation of the Rho-kinase pathway [104].

Studies investigating the relationship between t-PA, PAI-1 and SVD are limited and inconsistent. Lacunar stroke patients (n = 65) had higher levels of t-PA 1–3 months post-stroke compared to cortical stroke patients (n = 60) after adjusting for age, sex and vascular risk factors, perhaps indicating a role in the development or progression of mechanisms specific to SVD [70]. Dobrynina et al. [108] also reported a significant positive correlation between t-PA levels and WMH severity and PVS burden in 71 SVD patients and 21 healthy controls; however, they did not adjust for potential clinical covariates in this study. Conversely, a study with 100 patients of recent cortical or lacunar ischaemic stroke failed to find a significant relationship between plasma t-PA concentrations and PVS count or volume after adjusting for age, sex and vascular risk factors [73]. No association was found between PAI-1 levels and WMH volume or the presence of lacunes in 651 community-based elderly participants or in 71 SVD patients and 21 healthy controls [108,109].

#### 4.5.4. Prothrombin Factors 1 and 2

Prothrombin factors 1 and 2 are coagulation factors that can cleave to form thrombin, which is the key enzyme that transforms fibrinogen into fibrin and facilitates platelet activation. There was no reported association between circulating levels of prothrombin factor 1 and 2 and PVS in the basal ganglia in 100 ischaemic stroke patients, including lacunar stroke patients [73]. In the aforementioned Austrian Stroke Prevention Study, no significant associations were detected between prothrombin factors 1 and 2 and SVD features or WMH lesion progression at 3- or 6-year follow-up in 296 community-dwelling participants [50]. However, as participants in this study were asymptomatic, it remains a possibility that relationships between SVD and coagulation factors could go undetected in this cohort; subsequent power analyses also revealed larger sample sizes would be necessary to detect significant differences if a subtle effect size were to be assumed.

#### 4.5.5. D-Dimer

D-dimer is a by-product of blood clotting and fibrin degradation produced when fibrin is lysed with plasmin. High levels are indicative of plasmin activation and fibrin production and have been associated with cardiovascular disease and future atherothrombotic events [110]. There was no reported association between levels of D-dimer and PVS in the basal ganglia in 100 recent ischaemic stroke patients, including lacunar stroke patients [73]. There was also no relationship found between circulating levels of D-dimer and SVD features or progression in 296 community-dwelling participants in a 6-year longitudinal study [50]. Albeit perhaps unrelated to pathophysiological mechanisms specific to SVD, one study with 102 SVD patients did find a relationship between baseline serum concentrations of D-dimer and an increased risk of a cardiovascular event (including recurrent stroke) or death after adjusting for age, sex and radiological markers of SVD [71].

#### 4.5.6. Thrombomodulin

Thrombomodulin (TM) is a vasoactive factor expressed on the surface of endothelial cells. It engenders a powerful anticoagulation effect by decreasing circulating levels of thrombin and inhibiting factors Va and VIIIa through the production of activated protein C [107,111]. TM can reflect systemic endothelial damage, although some studies suggest it can protect the endothelium by attenuating inflammation-inducing injury [18,107]. Increased circulating TM concentrations were significantly associated with a higher number of lacunes and more severe WMH burden in 110 lacunar stroke patients, with higher TM levels reported in SVD patients compared to 50 healthy controls after adjusting for vascular risk factors [18]. Similarly, a cross-sectional analysis with 651 community-based ageing participants (average age: 67.4) demonstrated higher average serum concentration levels of TM present in those with lacunar infarcts or moderate WMH compared to those without [109]. In contrast, no association was found between levels of TM and WMH lesion progression at 6-year follow-up after adjusting for potential confounding variables in 296 community-dwelling participants in the Austrian Stroke Prevention Cohort [50].

Intriguingly, an ex vivo pathology study examining small penetrating cerebral arteries in 17 SVD cases, 12 aged controls and 4 young controls found significantly higher levels of TM immunoreactivity in SVD cases compared to aged controls and in vessels with a higher sclerotic index; TM was entirely absent or sparse in young controls [52].

#### 4.5.7. Endothelin-1

Endothelin-1 (ET-1) is a potent exogenous vasoconstrictor peptide produced by endothelial cells in response to hypoxia, shear stress, and exposure to cytokines, growth factors and thrombin. ET-1 can induce vasoconstriction, hypertrophy, fibrosis and increased BBB permeability and can also mediate the release of NO, implicating it in hypertension and endothelial dysfunction [107]. ET-1 has been associated with synaptic dysfunction and consequent cognitive deficits in pre-clinical investigations featuring a mouse model of transient vasoconstriction via bilateral injections of ET-1 [112]. Research examining the possible role of ET-1 in SVD is limited. A large (n = 1396) 6-year longitudinal study found baseline serum ET-1 levels to be positively associated with worsening WMH burden (Fazekas scale score ≥ 2) and the presence of lacunes and microbleeds after adjusting for age, sex, vascular risk factors, medication use, carotid stenosis and baseline WMH burden [46].

#### 4.5.8. Fibrinogen

Fibrinogen is a blood plasma glycoprotein produced in the liver. It is the main structural component of a blood clot and functions as a coagulation factor. Fibrinogen can also indicate capillary leakage and can disrupt BBB integrity by converting to toxic blood-derived fibrin deposits, which disrupt blood flow and result in downstream consequences affecting other constituents of the neurogliovascular unit, eventually compromising white matter integrity [113]. Participants with elevated fibrinogen levels tended to have a more severe WMH burden and a greater number of lacunes in a Japanese community-based neuroimaging study (n = 651) [114]. Logistic regression analyses adjusted for age, sex, vascular risk factors, CRP levels, and carotid artery atherosclerosis revealed an independent association between fibrinogen levels and WMH burden. In the same cohort, levels of fibrinogen were inversely correlated with cognitive performance, albeit only in patients without WMH. Findings from the Framingham Heart Study (n = 1763) did not support an association between circulating levels of fibrinogen and WMH volumes or the presence of lacunes or microbleeds [44]. Furthermore, there was no association between circulating levels of fibrinogen and basal ganglia PVS in 100 patients with recent ischaemic stroke [73].

### 4.6. Growth Factors

Vascular endothelial growth factor (VEGF), placental growth factor (PlGF) and transforming growth factor-β1 (TGF-β1) are potent angiogenic factors crucial for endothelial cell proliferation, migration, maturation and survival; they play a key role in angiogenesis, microvascular permeability and overall vessel function [115,116]. A fundamental feature of ageing involves angiogenic signalling failure and possible subsequent alterations in blood flow [117]. Mice with a genetic deletion of VEGF display endothelial cell degeneration and death [118]. There is evidence to suggest that abnormally raised or decreased levels of VEGF and TGF-β1 are associated with cardiovascular disease, perhaps via their induction through hypoxia-pertinent pathways or through stimulation of NO production [119]. There is also evidence to suggest VEGF and PlGF encourage vascular permeability and inflammation by uncoupling endothelial cell-to-cell junctions [120,121]. Injections of VEGF can result in fibrinogen and plasma clotting, increased BBB permeability and vascular inflammation [122].

Few clinical studies have investigated the link between growth factors and SVD. We identified eight studies with a total of 5176 participants (average sample size: 739.43).

In a cross-sectional study with 293 thrombolysed acute ischaemic stroke patients, higher serum VEGF levels at 90 days post-stroke, though not baseline levels at stroke onset, were associated with the presence of lacunes and greater global SVD disease burden on CT after adjusting for age, sex, and vascular risk factors; there was no association with WMH burden specifically [40]. One study reported higher VEGF levels in a group of 91 large vessel atherosclerotic disease patients compared to 89 SVD patients within 24 h of acute ischaemic stroke onset [123]. However, serum VEGF levels were proportional to infarct volume after adjusting for potential confounding variables, which might explain the increased levels in the group with strokes of large-artery aetiology, which have larger volumes. Group differences in VEGF levels were attenuated by 3 months post-stroke, though higher serum levels of VEGF in the acute stroke phase were associated with a better functional outcome (National Institute of Health Stroke Scale (NIHSS) score decrease > 4 points) after adjusting for potential covariates including initial NIHSS and infarct volume, possibly due to their neuroprotective and angiogenic effects. However, it is important to note that the well-established roles of VEGF in acute-ischaemic stroke render the extrapolation of findings from these populations arguably unreliable in the context of SVD, as significant fluctuations in growth factor levels after an acute ischaemic event cannot be discounted.

Some large studies in stroke-free ageing populations report no significant associations between VEGF and radiological markers of SVD. For instance, no connection was reported between circulating levels of VEGF and brain atrophy, WMH volume or white matter tract integrity (fractional anisotropy via Diffusion Tensor Imaging (DTI) sequence on MRI) in a young (average age: 46) subset of 1853 participants in the Framingham Heart Study cohort after adjusting for demographic and clinical variables [124]. Likewise, Shoamanesh et al. [44] did not observe an association between circulating VEGF levels and WMH burden, microbleeds or lacunes in 1763 stroke-free adults (average age: 60.2) in the Framingham offspring cohort.

In contrast, higher baseline levels of the VEGF family and PlGF in CSF were associated with greater WMH volume after adjusting for age, sex, ICV and vascular risk factors in 247 elderly participants with MCI (average age: 70.89), though not in 495 cognitively unimpaired participants; adjusting for CSF Aβ-40 attenuated the relationship for VEGF but not PlGF [49]. PlGF was amongst the strongest independent predictors of WMH at baseline in cognitively impaired and unimpaired individuals, independent of Aβ status, providing evidence of the overlapping implications between inflammation and cerebrovascular disease. Curiously, lower levels of VEGF and PlGF were associated with increasing WMH lesions over 7 years, leading the authors to hypothesise that vascular injury might inflict enough damage to endothelial cells to lead to a depletion in growth factor production and a subsequent decline in angiogenesis. Results from the ongoing MarkVCID consortium also indicate that in individuals at risk for cognitive impairment and dementia, PlGF may function as a candidate biomarker measuring the severity of vascular injury and WMH lesion burden and may serve as an accurate cross-sectional diagnostic biomarker and contribute as a prognostic susceptibility biomarker longitudinally [125]. In this study, Hinman et al. [125] observed a significant relationship between circulating plasma PlGF levels and WMH lesion burden (measured volumetrically and using the Fazekas scale) as well as cognitive impairment as measured by the Clinical Dementia Rating Scale (CDR) after adjusting for age and sex. Encouragingly, PlGF levels showed increasing diagnostic accuracy for all progressive disease states: WMH only (AUC: 0.66), mild cognitive impairment with WMH (AUC: 0.74) and dementia with WMH (AUC: 0.89). The authors posit that SVD mechanisms might be driving an increase in angiogenic factor circulation paired with a failure in downstream receptor signalling in cerebral microvessels, possibly involving Flt-1 receptor activity.

To determine whether the heterogeneity observed in the clinical and radiological manifestations of SVD could be attributed to different pathogeneses, Dobrynina et al. [81] categorised 40 SVD patients with a Fazekas score of three into two subgroups of radiological manifestations based on a cluster comparison and examined whether they were related to different blood biomarkers. Univariate analyses revealed lower levels of VEGF in patients in an MRI subgroup with widespread WMH and lacunes, microbleeds, atrophy and severe cognitive and gait impairments compared to 21 healthy controls or those in the other MRI subgroup, categorised by more severe deep WMH, lacunes in the white matter, no microbleeds or atrophy and less severe clinical manifestations, though the latter was not statistically significant. Whilst it is possible that VEGF could contribute to a specific SVD radiological profile amongst patients with severe WMH lesions, it is worth noting that these analyses did not include important clinical covariates that may influence blood biomarker concentrations, despite significant differences between SVD patients and controls. Notably, SVD patients were substantially more likely to be hypertensive (85.4% versus 43.5%) and diabetic (17.7% versus 0%).

In a 5-year longitudinal imaging study with 35 SVD patients, 43 age-matched participants with a history of stroke (further subdivided into lacunar and atherothrombotic) and 46 age-matched controls, TGF-β1 levels were highest among those with deep WMH lesions, which had progressed at 5-year follow-up [126]. Logistic regression analyses indicated that the odds ratio for the presence of elevated TGF-β1 was significant after adjusting for age, sex and major vascular risk factors. It is not entirely clear why this increase was not observed in the subset of participants with a history of lacunar stroke or why it was unrelated to periventricular WMH lesion presence and progression.

### 4.7. Nitric Oxide Pathways

#### 4.7.1. Nitric Oxide

NO, an endothelium-derived vasodilator, inhibits platelet activation and thrombosis and limits the adhesion of inflammatory cells. Dysfunctional endothelial cells decrease NO bioavailability by reducing NO synthesis and secretion, which can lead to defective vasodilatory processes, impaired blood flow, platelet aggregation, proliferation of smooth muscle cells and increased blood pressure [127,128]. In the endothelium, NO is produced by eNOS through calcium-dependent binding of calmodulin. Hence, intracellular calcium can influence eNOS and NO production. NO can alter the calcium (Ca^2+^) in smooth muscle cells through second messenger and potassium (K+) channel activation pathways that facilitate contraction and relaxation. Levels of endothelial-derived factors, including NO, must be tightly regulated to allow for healthy BBB and brain function [129,130]. A population-based longitudinal study (average follow-up: 69.7 months) with 1396 stroke-free adults reported that changes in NO levels were significantly negatively associated with an increased risk of new incident WMH lesions, lacunes, and microbleeds on MRI after adjusting for age, sex, vascular risk factors, medication, carotid stenosis and baseline WMH volume [46].

#### 4.7.2. NOSTRIN

Endothelial nitric oxide synthase traffic inducer (NOSTRIN) is involved in the regulation of perfusion and inflammation and is thought to inhibit the production of NO by sequestering and thus attenuating the activation of eNOS [131]. In addition, NOSTRIN may influence the expression of secreted cytokines and can restrict endothelial cell proliferation and adhesion [132]. Using EDE biomarkers, Elahi et al. [54] examined the cargo proteins within plasma membrane-derived vesicles of brain endothelial cells from 26 neurologically-normal older adults. They found higher levels of NOSTRIN in participants with WMH lesions compared to those without, though they did not find significant associations between NOSTRIN levels and global grey matter volumes or cognitive performance; it is important to note that only 11 patients within this small sample size had signs of SVD.

#### 4.7.3. ADMA

Asymmetric dimethylarginine (ADMA) is a nitric oxide synthase (NOS) inhibitor associated with traditional vascular risk factors, stroke, and endothelial dysfunction, presumably via a reduction in cerebral blood flow or the promotion of oxidative stress and inflammation [133]. NOS is an endogenous enzyme involved in the conversion of substrate L-arginine to NO, the former of which is involved in various processes crucial to the maintenance of cerebrovascular health, including vasodilation, platelet aggregation inhibition, smooth muscle cell generation and vascular cell adhesion. Due to their structural affinity, ADMA and L-arginine compete for NOS binding, hence reducing the bioavailability of NO [134].

Serum ADMA levels were significantly higher in a group of 210 SVD patients compared to 207 healthy controls; however, this difference was no longer significant when adjusting for potential confounders, including age, sex, vascular risk factors and coronary heart disease [135]. ADMA levels differed significantly between SVD-associated lesion types, with higher levels reported in patients with an isolated lacune compared to those with multiple visible lacunes and significantly lower levels in those with WMH lesions compared to those with WMH lesions and accompanying cerebral atrophy. Though the study sample size is relatively small, one possible explanation for these findings relates to the potential role of ADMA in the development of atherosclerosis, which might play a more direct role in isolated lacunar infarction. Though no significant correlation was found between levels of ADMA and cognitive impairment, subgroup analyses revealed that in patients with WMH lesions specifically, levels of ADMA were significantly correlated with Montreal Cognitive Assessment (MoCA) scores. These results suggest that though ADMA may not play a key role in instigating SVD pathophysiology, it could exacerbate disease progression, conceivably through the inhibition of NO production and consequent cerebral blood flow changes.

Khan et al. [136] observed higher levels of circulating ADMA in 47 SVD patients compared to 38 controls with similar vascular risk factor profiles. ADMA levels were not correlated with the number of lacunes, though WMH severity grade was positively correlated with ADMA (OR 10.04, 95% CI: 1.70–59.27) after controlling for age, sex, vascular risk factors and creatinine clearance. Though the authors hypothesised that ADMA might mediate the relationship between homocysteine (Hcy) and endothelial activation, they found no significant associations between ADMA and Hcy levels and the relationship between ADMA and SVD was only slightly attenuated after controlling for Hcy. Along the same vein, a study with young, asymptomatic patients (average age: 51.1) also reported significantly higher levels of ADMA amongst those with SVD compared to controls [137]. ADMA levels were positively correlated with WMH presence and severity after adjusting for potential confounding variables, further supporting the role of ADMA and endothelial dysfunction in early disease pathology.

#### 4.7.4. Myeloperoxidase

Myeloperoxidase is an oxidizing enzyme secreted by neutrophils, leukocytes and microglia following neuronal injury. It restricts NO bioavailability by consuming it and also contains hypochlorous acid, and can thus aggravate inflammation, incite tissue damage and has been associated with BBB and endothelial dysfunction [138]. In the Framingham Heart Study, circulating levels of myeloperoxidase were measured as a proxy biomarker for oxidative stress in 1763 stroke-free participants (average age: 60.2) [44]. After controlling for age, sex, vascular risk factors and time from blood draw to MRI (average: 5 years), they observed significantly higher levels of myeloperoxidase in the presence of microbleeds (OR 1.5, 95% CI: 1.1–2.0), with each additional unit increase resulting in a 1.9-fold increased risk of having more than one microbleed. The authors also reported a non-significant association between lower myeloperoxidase levels and the presence of WMH lesions and/or lacunes. It remains unclear why lower levels of myeloperoxidase were associated with ischaemic SVD-related lesions and higher levels with haemorrhagic disease manifestations, and it remains feasible that these might have distinct pathophysiological causes. In addition, it is possible that chronic SVD could lead to an overburdened inflammatory pathway, which might eventually deplete circulating myeloperoxidase availability.

### 4.8. Other Relevant Biomarkers

#### 4.8.1. Homocysteine

Hcy is a sulphur-containing protein usually excreted from the body via the kidneys. It is associated with cardiovascular events and stroke risk as well as cognitive impairment and is indicative of endothelial dysfunction [139,140]. Increased levels of Hcy are thought to exert deleterious effects on the endothelium and can facilitate oxidative stress, impair NO-mediated blood flow processes, up-regulate redox-sensitive inflammatory pathways, as well as promote atherogenesis, thrombogenicity and the aggregation of ADMA [141,142,143].

Higher levels of Hcy were reported in 74 lacunar stroke patients 1-month post-stroke compared to 74 healthy controls matched for age, sex, hypertension status and diabetes [38]. Though many inflammatory markers had normalised by 3 months post-stroke, Hcy levels remained unchanged, suggesting it was not a by-product of the acute ischaemic event itself. In a UK-based study of 457 black stroke patients and 179 black community controls, elevated serum Hcy concentrations were observed in SVD patients compared to controls after adjusting for age, sex, vascular risk factors and renal function [144]. In addition, Hcy levels within SVD patients were positively correlated with WMH severity and were associated with the presence of lacunes with confluent WMH lesions. Hcy levels were also found to be independently associated with a greater WMH volume burden after adjusting for demographics and vascular risk factors in 809 acute ischaemic stroke patients (average age: 65.6), with each quartile increase in plasma Hcy being associated with a WMH volume increase of 11% [145]. Furthermore, Hcy levels were positively correlated with the number of lacunes (OR 2.14, 95% CI: 1.4–3.27) in a large population-based cohort study of 1023 participants (average age: 56.7) after adjusting for age, sex and vascular risk factors [146]. Another study also reported higher circulating Hcy in 47 SVD patients compared to 38 healthy controls with similar risk factor profiles, which were positively correlated with WMH severity grade and number of lacunes after controlling for demographic and clinical covariates as well as creatinine clearance [136].

In a large Chinese community-based cohort study (n = 960, average age: 56.02), grouped endothelial dysfunction biomarkers, including Hcy, were independently associated with WMH volume and number of lacunes, though not microbleeds or PVS; backward elimination regression retained Hcy as an independent risk factor for the presence of lacunes (OR: 1.44, 95% CI: 1.20–1.74) [43]. Hcy levels were higher in a group of 172 SVD patients compared to 172 age- and sex-matched controls [147]. After controlling for traditional vascular risk factors and age, Hcy was also a strong risk factor in those with WMH lesions. Consistent with the hypothesis that Hcy mechanistically relates to SVD through endothelial dysfunction, the authors found that including other markers of endothelial dysfunction, ICAM-1 and TM, in a logistic regression model resulted in the loss of the significant association between Hcy and SVD.

Results from the Framingham Heart Study (sample size: 1763 stroke-free participants) contradict these findings, and the authors report no association between circulating Hcy levels and several markers of SVD on MRI, including WMH lesions, lacunes and microbleeds [44].

Some studies also suggest a possible association between Hcy and clinical outcome or disease progression. For instance, in a cohort of 123 SVD patients (average age: 66.4), circulating Hcy levels correlated with WMH severity, number of lacunes, basal ganglia PVS and lobar microbleeds, and also served as an independent predictor for global cognitive impairment [148]. Hcy was also associated with radiological progression of SVD at a 2-year follow-up in a longitudinal cohort study with 123 SVD patients, with higher circulating levels related to the development of new lacunes as well as an increased risk of a recurrent vascular event or death after controlling for demographic variables, vascular risk factors and disease burden [47,71]. Interestingly, a recent study reported significantly higher levels of plasma Hcy in SVD patients with cognitive impairment (n = 40) compared to SVD patients without cognitive impairment (n = 38) and healthy controls (n = 35), respectively; however, potential confounding variables were not adjusted for in these univariate analyses [90].

#### 4.8.2. Albumin

Microalbuminaria (urinary albumin excretion < 20–200 µg/min) is associated with systemic dysfunction of the vascular endothelium, elevated levels of fibrinogen, BBB dysfunction and an increased risk of stroke [149,150,151]. Though the precise mechanisms underlying these associations remain mostly unknown, it is possible that cytokines and other factors implicated in inflammation might mediate renal and glomerular damage, contributing to albuminaria and SVD [152]. Furthermore, small vessels in the brain and the kidneys share haemodynamic and physiological similarities and damage to the kidney endothelium can incite albumin leakage [153]. It is also plausible that this relationship is mediated by the involvement of relevant risk factors that might also influence renal function, such as sodium consumption, diabetes and hypertension.

In a Japanese community-based study with 651 participants (average age: 67.4), those with albuminaria had higher WMH grades and the total number of lacunes compared to those without; in addition, the total number of lacunes and WMH severity grade were significantly higher in the highest tertile of albumin–creatinine ratio [109,154]. A standard deviation increase in the albumin–creatinine ratio was associated with a nearly 2-fold increased risk of having at least one lacunar infarct and a 2.15-fold increased risk of the presence of WMH lesions, independent of age, sex and vascular risk factors.

In the longitudinal Norwegian HUNT2 population-based study with 7261 participants, albuminaria was associated with increased overall ischaemic stroke risk over a median follow-up period of 15 years [155]. This association was modestly stronger for the lacunar subtype compared to large-artery or cardioembolic ischaemic stroke subtypes or haemorrhagic stroke, even after controlling for age, sex and vascular risk factors, perhaps providing further evidence for the role of albuminaria in diffuse endothelial and vascular dysfunction in SVD [155].

#### 4.8.3. Haemoglobin A1c

HbA1c is a metabolite that functions as an indicator of chronically elevated glucose levels or insulin resistance and a biomarker of endothelial dysfunction. HbA1c is related to inflammation, the production of atheroma, thrombogenicity and the promotion of oxidative stress through the facilitation of ADMA synthesis [133,156]. In 809 acute ischaemic stroke patients (average age: 65.6), HbA1c levels were associated with WMH volumes on MRI and functioned as an independent predictor for WMH volume after adjusting for age, sex, race, vascular risk factors (including diabetes), coronary artery disease and prior stroke [145]. A quartile increase in HbA1c levels was associated with a 10% increase in WMH lesion burden.

#### 4.8.4. Amyloid-Beta

β-amyloid_1–40_ is associated with cerebral amyloid angiopathy and has been shown to alter endothelial cell-mediated cerebral blow flow regulation in mice, possibly through the formation of ROS, which can further harm the endothelium [157]. In 149 acute lacunar stroke patients and 25 age-matched controls, higher levels of plasma β-amyloid_1–40_ were reported in SVD patients with WMH lesions and multiple lacunar infarcts with or without the presence of WMH, compared to those with an isolated lacunar infarct in the absence of WMH or healthy controls [158]. The authors attribute this difference to potentially distinct disease pathologies, a diffuse arteriopathy versus localised vessel atheroma, with endothelial dysfunction playing a more prominent role in the former.

#### 4.8.5. Adiponectin

Adiponectin, a protein synthesised by adipose tissue, maintains vasoprotective properties by attenuating both inflammation and the production of ROS whilst enhancing NO generation and promoting the survival of endothelial progenitor cells [159]. The potentially protective role of adiponectin was demonstrated in a study with 133 patients with first-ever ischaemic stroke, where circulating levels of adiponectin were inversely associated with the prevalence of cerebral microbleeds after adjusting for age, sex and cardiovascular risk factors [37].

#### 4.8.6. Klotho

Predominantly produced by the kidneys, the Klotho proteins are components of endocrine fibroblast growth factor receptor complexes that mediate metabolic processes. With circulating levels decreasing with age, Klotho is often referred to as having ‘anti-ageing’ properties due to its involvement in the regulation of oxidative stress, organ protection and the inhibition of cellular senescence [160]. In a prospective study with 262 first-ever ischaemic stroke patients (average age: 67.4), plasma Klotho concentration was inversely associated with the presence of WMH lesions, PVS, lacunes, total SVD burden and disease progression, though not microbleeds, possibly suggesting different pathological mechanisms underlying the protective involvement of Klotho proteins in distinct radiologic manifestations of SVD [161]. How Klotho could exert its protective qualities on the cerebrovascular system is unclear, though the regulation of NO production and bioavailability in vascular endothelial cells remains a possible mechanism.

#### 4.8.7. Lipoprotein-Associated Phospholipase A_2_ Mass

Lipoprotein-associated phospholipase A_2_ mass (Lp-PLA_2_), an enzyme secreted by macrophages, serves to hydrolyse oxidised phospholipids and functions as a marker of vascular inflammation. Previous studies have suggested an association between high levels of Lp-PLA_2_ and stroke or SVD [162,163]. In addition, a relationship between Lp-PLA_2_ and dilation in the retinal venules was reported in the Rotterdam cohort, further substantiating its involvement in small vessel pathology [164].

In 1763 stroke-free participants in the Framingham Heart Study, Shoamanesh et al. [44] observed higher levels of circulating Lp-PLA_2_ in participants with lacunes and greater WMH volumes, as well as a non-significant trend between higher levels of Lp-PLA_2_ and the increasing number of microbleeds after adjusting for age, sex and vascular risk factors. As part of the Northern Manhattan Study, Wright et al. [165] similarly reported an association between higher levels of Lp-PLA_2_ and a higher WMH burden in 527 participants (average age: 71.3), with those in the highest quartile having a 1.3-fold increase in WMH volume compared to those in the lowest quartile, independent of demographic variables, the presence of risk factors, or other markers of inflammation.

## 5. Conclusions and Future Perspectives

**Figure 2:**
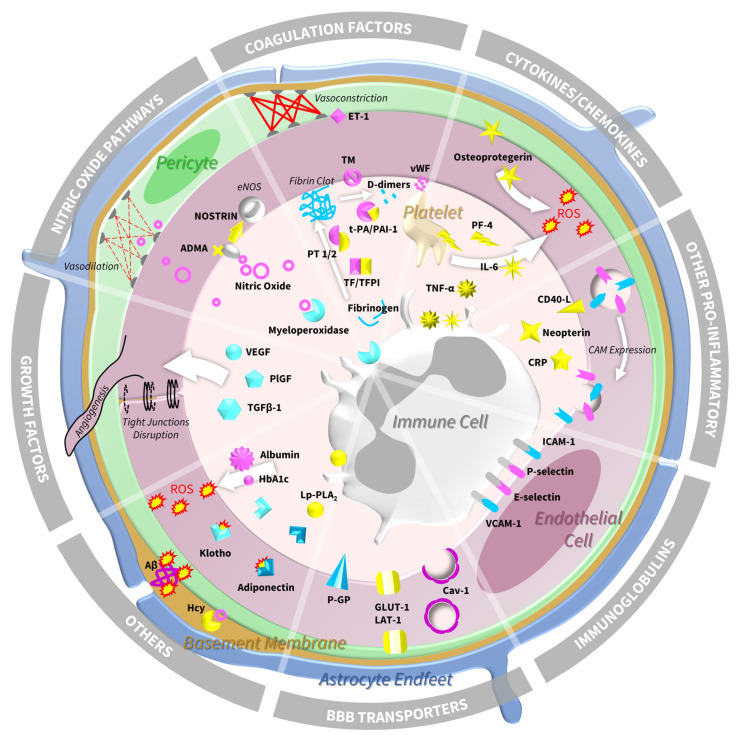
Biomarker roles in the gliovascular unit. Each octant represent a subdivision used in the review. Abbreviations per octant: **(Coagulation Factors)** ET-1: endothelin-1; TM: thrombomodulin; t-PA: tissue-type plasminogen activator; PAI-1: plasminogen activator inhibitor-1; PT 1/2: prothrombins 1 and 2; TF: tissue factor; TFPI: tissue factor pathway inhibitor; vWF: von willebrand factor; **(Cytokines/Chemokines)** ROS: reactive oxygen species; PF-4: platelet factor-4; IL-6: interleukin-6; TNF-α: tumour necrosis factor alpha; **(Other Pro-Inflammatory)** CD40-L: CD40-ligand; CRP: C-reactive protein; CAM: cell adhesion molecules; **(Immunoglobulins)** ICAM-1: intracellular cell adhesion molecule-1; P-selectin: platelet selectin; E-selectin: endothelial selectin; VCAM-1: vascular cell adhesion molecule-1; **(BBB Transporters)** Cav-1: caveolin-1; GLUT-1: glucose transporter-1; LAT-1: large neutral aminoacid transporter-1; P-GP: permeability-glycoprotein; **(Others)** Hcy: homocysteine; Aβ: amyloid beta; Lp-PLA_2_: lipoprotein-associated phospholipase A_2_-mass; HbA1c: haemoglobin A1c; **(Growth Factors)** VEGF: vascular endothelial growth factor; PlGF: placental growth factor; TGFβ-1: transforming growth factor beta-1; **(Nitric Oxide Pathway)** NO: nitric oxide; eNOS: endothelial nitric oxide synthase; ADMA: asymmetric dimethylarginine; NOSTRIN: endothelial nitric oxide synthase traffic inducer (schematic is a theoretical representation and is not drawn to scale).

In this scoping review, we have summarised findings from clinical neuroimaging studies investigating the relationship between radiological indications of SVD and biochemical markers of endothelial dysfunction in blood or CSF with the overarching aim of conceptualising the molecular mechanisms underlying the role of endothelial dysfunction in SVD (Figure 2).

Overall, there is evidence of an association between E-selectin and the presence of microbleeds as well as VCAM-1, ICAM-1 and P-selectin levels with WMH lesion severity and progression stemming from large community-dwelling cohort studies as well as cross-sectional and longitudinal studies with SVD patients, even when compared to cortical stroke patient controls.

Clinical research studies investigating the role of BBB transport proteins in SVD pathology are scarce, but we describe some studies that suggest a relationship between higher levels of GLUT-1, LAT-1 and P-GP and increasing WMH lesion burden as well as lower levels of Cav-1 and the presence of microbleeds.

Results from studies linking cytokine and chemokine biomarkers to SVD pathogenesis are inconclusive, and while there is evidence of a relationship between higher IL-6 levels and SVD burden, studies comparing patients of acute lacunar stroke with patients of acute thromboembolic occlusive stroke indicate a possible mediating role of an acute endothelial response to injury. Nonetheless, there is some published evidence in support of the role of osteoprotegerin, TNF-α and PF-4 in SVD mechanisms.

The contribution of inflammatory pathways to SVD pathogenesis remains elusive. In some large population-based studies, CRP levels were independent predictors of WMH lesion burden and progression as well as the number of lacunes or lacunar infarcts, though this was not the case in all the studies described. Indeed, most studies featuring acute and subacute stroke patients as controls did not observe strong associations between CRP levels and SVD burden. There is some evidence linking neopterin, though not CD40 ligand, to SVD.

Several lines of evidence also point to dysregulation in the nitric oxide pathway as being an important event in the pathogenesis of SVD. Findings from several studies suggest associations between levels of NO, NOSTRIN, ADMA and myeloperoxidase and the presence and degree of diverse SVD lesion types.

We did not find consistent evidence advocating for the role of activation of coagulation in the development and progression of SVD, with most studies evaluating a connection between vWF and SVD reporting negative results and those focussing on TFPI, t-PA, PAI-1, prothrombin factors 1 and 2, D-dimer and fibrinogen reporting contradicting results. However, some studies did report significant associations between higher levels of circulating TM and ET-1 and SVD severity.

Growth factors have been shown to have neuroprotective and angiogenic properties, but they also can promote vascular permeability, exacerbate ischaemic tissue injury post-stroke and lead to aberrant angiogenesis. Data on their influence over SVD mechanisms are inconsistent, particularly in studies with stroke populations at different post-stroke stages and of diverse aetiologies. Larger ageing cohort studies suggest there is little to no association between VEGF levels and SVD burden; however, other studies indicate an association with higher levels of other growth factors, notably TGF-β1 and PlGF, and WMH lesion burden and longitudinal disease progression.

We also found several studies supporting associations between levels of albumin, Hcy, HbA1c, β-amyloid_1–40_, Lp-PLA_2_ and adiponectin and several types of SVD lesions on MRI (Table 1).

Overall, the findings from this review point to a pivotal role of endothelial dysfunction in the molecular mechanisms underpinning SVD. Whilst there is no single definitive biomarker with consistent associations to specific clinical indicators of disease, promising independent predictors of disease progression include cell adhesion molecules, molecules pertaining to the nitric oxide pathway, PlGF, TGF-β1, and Hcy. It is possible that there are many ways in which endothelial dysfunction can exert influence over the development and progression of SVD pathophysiology. Although the initial causes of endothelial dysfunction remain unclear, there is a propensity towards a pro-inflammatory and perhaps a pro-thrombotic state accompanied by aberrant angiogenesis, microvessel luminal narrowing, reduced responsiveness (vasoreactivity), altered pulsatility (vascular stiffness) and vasomotion, increased BBB permeability, and impaired blood flow regulation. It is possible to conceive of these processes as a dynamic pathological cycle resulting in a downstream cascade of dysfunction involving the entirety of the neurogliovascular system and culminating in white matter injury and ischaemia, neurodegeneration, diffuse connectivity problems and cortical thinning.

### 5.1. Implications for Clinical Practice

A significant challenge impeding the progress of therapeutics for SVD stems from the lack of elucidative and highly specific biomarkers associated with disease development and progression. As it stands, and despite strong evidence advocating for the involvement of endothelial dysfunction in disease pathogenesis, there is not one biomarker that is capable of defining SVD or effectively adding a diagnostic or outcome-predictive ability in clinical practice. In large part, this is likely due to the complexity inherent in SVD mechanisms. SVD is a diffuse, chronic, and dynamic disease process marked by fluctuating radiological lesions and symptoms [166,167,168]. It is influenced by a complex interplay between genetic factors, lifestyle variables and environmental exposures, and although it is not predominantly an atherosclerotic disease, it shares an overlap in risk factors, mechanistic pathways, and indeed biomarkers with large-artery and cardiovascular disease. Our understanding of disease mechanisms is further hindered by the fact that human brain microvessels are not themselves visible on high-field MRI in vivo, and the generation of SVD-relevant animal models can be conceptually challenging. It is possible, and indeed likely, that there are many distinct routes through which endothelial dysfunction can drive SVD pathophysiology, and thus, it is reasonable to consider that a panel of biomarkers, rather than a sole molecule or pathway, is more likely to yield a comprehensive and robust diagnostic and prognostic tool in future.

### 5.2. Implications for Future Research

Another important reason a biomarker signature for SVD remains illusory pertains to the substantial inconsistencies in study results precipitated by heterogeneity in study design. There are several sources of methodological disparity impeding result replication and consistency. For instance, differences arise from the imaging modalities used, namely CT or MRI, and the methods and tools used to quantify radiological markers of disease. Many studies rely on diverse severity and grading scales, such as the Fazekas scale, to measure WMH lesion burden, foregoing the enhanced accuracy provided by volumetric quantification in sensitive analyses such as these. Studies also vary regarding the time window between serology and MRI acquisition, or the time between stroke onset and imaging/serology (when applicable). Another factor to consider is the variation introduced by the analytes themselves; this includes taking into account their stability, the measurement techniques used to quantify them and the presence of metabolic confounders (e.g., fasting status), comorbid illness (e.g., chronic kidney disease), or acute injury, infection, or ischaemia.

Another such source of heterogeneity relates to lesion types- whilst most studies focus on WMH (which are the most commonly observed SVD lesions), other related features, including lacunes, microbleeds, siderosis, brain atrophy, and PVS, remain understudied. It is possible that different lesion types arise at different disease stages, and with SVD representing a dynamic process, considering cumulative and total SVD burden rather than individual lesion types would likely prove insightful. However, perhaps the most critical source of variation comes from the characteristics of the study populations. Large ageing or community-dwelling cohort studies often deliberately will exclude patients with a history of stroke or MCI/dementia and tend to feature few patients with severe or even symptomatic SVD, limiting the representation of the full spectrum of disease and lowering their statistical power to detect significant or meaningful associations between biomarkers and clinical indicators of disease. There is also considerable variation in population characteristics amongst studies with SVD patients, with some including solely hypertensive patients, diabetic patients or indeed excluding patients with overt clinical manifestations of SVD, including stroke or cognitive impairment. In this context, it also remains crucial to consider the characteristics of the control population and the importance of controlling for potential demographic and clinical covariates. Comparing vascular function biomarkers between recent lacunar stroke patients and healthy controls introduces the likelihood that differences identified are influenced by the recent ischaemic event (i.e., markers of acute brain injury) or a greater burden of vascular risk factors in the former group.

Finally, it is worth addressing the limitations inherent in cross-sectional studies in terms of providing conclusions regarding the causal relationship between fluid biomarkers and SVD mechanisms. Large longitudinal studies, particularly in conjunction with advanced MRI techniques that allow for insight into BBB function and vascular functionality, such as dynamic contrast-enhanced (DCE) MRI and cerebrovascular reactivity (CVR), can be advantageous by helping to provide support for a causal role or delineating predictive effects.

Many important questions remain. Importantly, the extent to which these biomarkers accurately reflect endothelial dysfunction confined to the brain (as opposed to systemic dysfunction), or whether endothelial dysfunction in SVD is indeed confined to the brain, remains unclear. In addition, the many ways in which endothelial dysfunction could affect other constituents in the neurogliovascular system and how changes in these cells could, in turn, affect the endothelium remains poorly understood. Finally, candidate catalysts for endothelial dysfunction in the context of SVD and how they relate to genetics and vascular risk factor exposure remain unknown and warrant further examination.

There is substantial evidence proposing endothelial dysfunction as a pivotal mechanism in the pathogenesis of SVD, and the identification of relevant biomarkers and their association with disease mechanisms not only provides important clues regarding the different pathways involved, but it can also provide promising avenues for therapeutic targets. Particularly exciting prospects include clinical trials targeting endothelial dysfunction, including the ongoing LACI-2 trial, which is repurposing Isosorbide Mononitrate and Cilostazol as potential treatments for patients with symptomatic SVD [169].

## Figures and Tables

**Table 1:** Associations between individual biomarkers and distinct radiological manifestations of SVD present in the literature reviewed.

Class	Biomarkers	Role in Endothelial (Dys) Function	Relation to SVD	Refs.
Cell adhesion molecules and selectins	VCAM-1 ICAM-1 P-selectin E-selectin	expressed by a cytokine-activated endothelium facilitate the recruitment, rolling, adhesion and transendothelial migration of leukocytes through the endothelium of vascular walls have been linked to ageing, BBB breakdown and age-related neurodegenerative disorders	higher levels found in SVD patients positively associated with disease burden and progression, worse clinical outcomes and cognitive decline positively associated with WMH and the prevalence of lacunes and microbleeds, though not PVS	[18,22,34,37,38,40,41,42,43,44,45,46,47,48,49,50,51,52,54,70,73]
BBB Transporters	GLUT-1 P-GP LAT-1 Cav-1	expressed by brain endothelial cells mediate the influx and efflux of solutes from the circulation through endothelial cells and into the brain and vice-versa implicated in BBB permeability, vessel tone and metabolic dysregulations change with ageing, possibly disturbing neurovascular coupling processes based on metabolic demand	higher levels found in SVD patients positively associated with WMH and lower cognitive performance scores and inversely associated with total grey matter volume Cav-1 levels inversely associated with the prevalence of microbleeds	[54,62]
Cytokines and chemokines	IL-6 TNF-α/TNFR2 Osteoprotegerin PF-4	pro-inflammatory molecules that can incite platelet production and engender endothelial dysfunction through an increase in Angiotensin II type 1 (AT1) receptor activity, an interaction with tumour necrosis factor alpha (TNF-α), and the induced production of reactive oxygen species (ROS), which can cause enhanced vascular superoxide production, exacerbate oxidative stress, and impair endothelium-dependent vasodilation associated with inflammation, stroke, atherosclerosis and cardiovascular events	higher levels found in SVD patients positively associated with presence of WMH, subcortical infarcts, lacunes, and microbleeds, though not PVS contradictory results regarding the association with disease progression and worse clinical outcomes, though strong associations reported regarding adverse cardiovascular events, including recurrent stroke some results possibly mediated by recent stroke	[43,44,45,47,48,49,68,69,70,71,72,73,81]
Other pro-inflammatory markers	CRP Neopterin CD40-L	these pro-inflammatory molecules can inhibit angiogenesis, promote atherosclerosis, mediate circulating cytokine levels, inhibit NO, decrease endothelial nitric oxide synthase (eNOS) activity in endothelial cells, increase Endothelin-1 production, attenuate endothelial-dependent vasodilation and increase endothelial cell adhesion molecule expression	most studies featuring acute and sub-acute stroke patients do not report strong associations between CRP levels and SVD disease burden contradictory results, with some showing a positive association with the prevalence of lacunar infarcts, PVS, WMH lesion burden and progression, though not microbleeds positively associated with the risk of recurrent stroke	[34,38,43,44,47,51,68,69,70,71,72,73,90,91,92]
Coagulation factors	vWF TF TFPI t-PA PAI-1 Prothrombin factors 1 and 2 D-dimer TM ET-1 fibrinogen	endothelial cells play a crucial role in the maintenance of physiological haemostasis and the modulation of thrombosis through the tight control of both pro-coagulant and anti-coagulant mechanisms the endothelium provides a surface for thrombosis formation, regulates blood flow and mediates the expression of vasoactive factors relating to platelet reactivity, coagulation and fibrinolysis	vWF levels are comparable between lacunar and cortical stroke patients at sub-acute stroke stages there are contradicting results regarding associations between TF/TFPI, t-PA/PAI-1 and fibrinogen and SVD mechanisms no associations reported with prothrombin factors 1 and 2 or D-dimer levels of TM are positively associated with WMH lesion severity and number of lacunes, though not disease progression ET-1 is positively associated with worsening WMH burden and the presence of lacunes and microbleeds	[18,38,40,46,50,52,70,71,73,99,108,109,114]
Growth factors	VEGF TGF-β1 PlGF	potent angiogenic factors crucial for endothelial cell proliferation, migration, maturation and survival play a key role in angiogenesis, microvascular permeability (possibly by uncoupling endothelial cell junctions), inflammation and overall vessel function a fundamental feature of ageing involves angiogenic signalling failure and alterations in blood flow associated with cardiovascular disease, perhaps through the induction of hypoxia-pertinent pathways or involvement of NO production	positively associated with the presence of lacunes and greater global SVD disease higher levels associated with better functional outcome post-stroke positively associated with greater WMH volume at baseline and inversely associated with WMH lesion progression longitudinally no associations reported with brain atrophy, white matter tract integrity or microbleeds	[40,44,49,81,123,124,125,126]
Nitric oxide pathway	NO NOSTRIN ADMA Myeloperoxidase	dysfunctional endothelial cells decrease NO bioavailability by reducing NO synthesis and secretion, which can lead to oxidative stress, inflammation, defective vasodilatory processes, impaired blood flow, platelet aggregation, proliferation of smooth muscle cells and increased blood pressure	NO levels are inversely associated with SVD progression and WMH, lacunes and microbleeds NOSTRIN levels are positively associated with WMH, though not grey mater volumes or cognitive performance ADMA levels are higher in SVD patients and positively associated with WMH, though not number of lacunes Myeloperoxidase levels are higher in SVD patients and positively associated with microbleeds, though not WMH or lacunes	[44,46,54,135,136,137]
Other relevant biomarkers	Hcy Albumin HbA1c β-amyloid_1–40_ Adiponectin Klotho Lp-PLA_2_	these molecules modulate a wide range of functions. Some exert deleterious effects on the endothelium and can facilitate oxidative stress, impair NO-mediated blood flow processes, up-regulate redox-sensitive inflammatory pathways, and promote atherogenesis and thrombogenicity, whilst others might offer protective neurovascular mechanisms	Hcy levels are higher in SVD patients and are positively correlated with WMH severity, number of lacunes, microbleeds, PVS, disease progression and cognitive impairment, though results are inconsistent albuminaria is associated with WMH severity, prevalence of lacunes and recurrent lacunar stroke HbA1c is positively associated with WMH volume increase β-amyloid_1–40_ levels are higher in those with WMH lesions and multiple lacunar infarcts levels of adiponectin are inversely associated with the prevalence of microbleeds Klotho levels are inversely associated with WMH, PVS, lacunes and total SVD burden and progression, though not microbleeds Lp-PLA_2_ levels are positively associated with WMH, lacunes and microbleeds	[37,38,43,44,47,71,90,109,136,144,145,146,147,148,155,158,161,165]

Abbreviations: Cell adhesion molecules: VCAM-1: vascular cell adhesion molecule-1, ICAM-1: intracellular cell adhesion molecule-1, P-selectin: platelet selectin, E-selectin: endothelial selectin. BBB transporters: GLUT-1: glucose transporter-1, P-GP: permeability-glycoprotein, LAT-1: large neutral amino acid transporter-1, Cav-1: caveolin-1. Cytokines and Chemokines: IL-6: interleukin-6, TNF-α: tumour necrosis factor alpha, TNFR2: tumour necrosis factor receptor 2, PF-4: platelet factor-4. Other pro-inflammatory markers: CRP: C-reactive protein, CD40-L: CD40 ligand. Coagulation factors: vWF: von Willebrand Factor, TF: tissue factor, TFPI: tissue factor pathway inhibitor, t-PA: plasminogen activator, PAI-1: plasminogen activator inhibitor-1, TM: thrombomodulin, ET-1: endothelin-1. Growth factors: VEGF: vascular endothelial growth factor, TGF-β1: transforming growth factor-β1, PlGF: placental growth factor. Nitric oxide pathways: NO: nitric oxide, NOSTRIN: endothelial nitric oxide synthase traffic inducer, ADMA: asymmetric dimethylarginine. Other relevant markers: Hcy: homocysteine, HbA1c: haemoglobin A1c, β-amyloid_1–40_: beta-amyloid, Lp-PLA_2_: lipoprotein-associated phospholipase A_2_-mass.

## Abbreviations

AD	Alzheimer’s Disease
ADMA	asymmetric dimethylarginine
AT1	angiotensin II type 1 receptor
Aβ	amyloid beta
β-amyloid_1–40_	beta-amyloid_1–40_
BBB	blood–brain barrier
Ca^2+^	calcium
CADASIL	cerebral autosomal dominant arteriopathy with subcortical infarcts and leukoencephalopathy
Cav-1	caveolin-1
CD40	CD40 ligand
CDR	Clinical Dementia Rating Scale
CRP	C-reactive protein
CSF	cerebrospinal fluid
CT	computerised tomography
CVR	cerebrovascular reactivity
DCE-MRI	dynamic contrast-enhanced MRI
DTI	diffusion tensor imaging
EDE	endothelial-derived exosome
eNOS	endothelial nitric oxide synthase
E-selectin	endothelial selectin
ET-1	endothelin-1
GLUT-1	glucose transporter-1
Hcy	homocysteine
HbA1c	haemoglobin A1c
ICAM-1	intracellular cell adhesion molecule-1
ICV	intracranial volume
IL-6	interleukin-6
K^+^	potassium
LAT-1	large neutral amino acid transporter-1
Lp-PLA_2_	lipoprotein-associated phospholipase A_2_-mass
MCI	mild cognitive impairment
MRI	magnetic resonance imaging
NO	nitric oxide
NOS	nitric oxide synthase
NOSTRIN	endothelial nitric oxide synthase traffic inducer
PAI-1	plasminogen activator inhibitor-1
PF-4	platelet factor-4
P-GP	permeability-glycoprotein
PlGF	placental growth factor
P-selectin	platelet selectin
PVS	perivascular spaces
ROS	reactive oxygen species
sVCAM-1	soluble vascular cell adhesion molecule-1
SVD	small vessel disease
TF	tissue factor
TFPI	tissue factor pathway inhibitor
TGF-β1	transforming growth factor- β 1
TM	thrombomodulin
TNFR1	tumour necrosis factor receptor 1
TNFR2	tumour necrosis factor receptor 2
TNF-α	tumour necrosis factor alpha
t-PA	tissue-type plasminogen activator
VCAM-1	vascular cell adhesion molecule-1
VEGF	vascular endothelial growth factor
vWF	von Willebrand Factor
WMH	white matter hyperintensities
